# MicroRNA-4719 and microRNA-6756-5p Correlate with Castration-Resistant Prostate Cancer Progression through Interleukin-24 Regulation

**DOI:** 10.3390/ncrna5010010

**Published:** 2019-01-21

**Authors:** Dibash K. Das, Leah Persaud, Moira Sauane

**Affiliations:** 1 Department of Biological Sciences, Herbert H. Lehman College, City University of New York, 250 Bedford Park Boulevard West, Bronx, NY 10468, USA; dibashdas486@gmail.com (D.K.D.); leah.persaud@lehman.cuny.edu (L.P.); 2 Department of Biology, The Graduate Center, City University of New York, 365 Fifth Avenue, Room 4315, New York, NY 10016, USA

**Keywords:** microRNA, castration-resistant prostate cancer, Interleukin-24, racial disparity

## Abstract

Prostate cancer (PCa) is the second leading cause of cancer death in the United States. The five-year survival rate for men diagnosed with localized PCa is nearly 100%, yet for those diagnosed with aggressive PCa, it is less than 30%. The pleiotropic cytokine Interleukin-24 (IL-24) has been shown to specifically kill PCa cells compared to normal cells when overexpressed in both in vitro and in vivo studies. Despite this, the mechanisms regulating IL-24 in PCa are not well understood. Since specific microRNAs (miRNAs) are dysregulated in PCa, we used miRNA target prediction algorithm tools to identify miR-4719 and miR-6556-5p as putative regulators of IL-24. This study elucidates the expression profile and role of miR-4719 and miR-6756-5p as regulators of IL-24 in PCa. qRT-PCR analysis shows miR-4719 and miR-6756-5p overexpression significantly decreases the expression of IL-24 in PCa cells compared to the negative control. Compared to the indolent PCa and normal prostate epithelial cells, miR-4719 and miR-6756-5p are significantly overexpressed in castration-resistant prostate cancer (CRPC) cell lines, indicating that their gain may be an early event in PCa progression. Moreover, miR-4719 and miR-6756-5p are significantly overexpressed in the CRPC cell line of African-American males (E006AA-hT) compared to CRPC cell lines of Caucasian males (PC-3 and DU-145), indicating that miR-4719 and miR-6756-5p may also play a role in racial disparity. Lastly, the inhibition of expression of miR-4719 and miR-6756-5p significantly increases IL-24 expression and inhibits proliferation and migration of CRPC cell lines. Our findings indicate that miR-4719 and miR-6756-5p may regulate CRPC progression through the targeting of IL-24 expression and may be biomarkers that differentiate between indolent and CRPC. Strategies to inhibit miR-4719 and miR-6756-5p expression to increase IL-24 in PCa may have therapeutic efficacy in aggressive PCa.

## 1. Introduction

Prostate cancer (PCa) is the most common non-skin cancer and the second leading cause of cancer-related death for men in the US [1,2,3]. Accounting for 180,890 new cases in the United States in 2016, PCa is a major cause of cancer morbidity and mortality [1,2,3]. The annual morbidity grows as the rate of PCa has increased by 14% over the last two decades [4,5,6]. Despite this, men with PCa do not die from localized prostate cancer but instead, castration-resistant prostate cancer (CRPC) [4,5,7]. In the US, the average 5-year survival rate for localized PCa is 100%, but for CRPC it is less than 30% [7,8,9]. Prostate specific antigen (PSA), the current tool for screening for PCa, is highly controversial because it is not PCa-specific and has a high false positive rate [7,8,9,10]. Consequently, new biomarkers are urgently needed for the early detection of PCa, and it is crucial that these novel biomarkers are sensitive enough to discriminate between indolent PCa and CRPC. 

Interleukin-24 (IL-24), also known as melanoma differentiation associated gene-7, is a multifunction cytokine that has been studied for its specific anti-cancer properties. Along with killing other cancer cell types, IL-24 induces apoptosis in prostate cancer cells through endoplasmic reticulum (ER) stress, mitochondrial dysfunction, reactive oxygen species accumulation, and downregulation of anti-apoptotic proteins [11,12,13,14,15,16]. In combination with treatments such as ionizing radiation and Sabutoclax, IL-24 can also sensitize prostate cancer tumors to apoptosis [17,18]. While several *in vitro* and *in vivo* studies have characterized IL-24 as specific cancer killing protein in PCa cells compared to normal prostate epithelial cells, IL-24 expression in PCa, specifically in CRPC cells, is not fully understood [11,12,15,17,19,20,21,22,23,24,25,26,27]. Here, we seek to understand the factors that may increase IL-24’s short mRNA half-life leading to the upregulation of IL-24 protein and thus, increased apoptosis in PCa and CRPC. 

MicroRNAs (miRNAs) are 20-24-nucleotide-short RNAs that play pivotal roles in almost all biological processes in mammalian species [28,29,30]. It is well established that miRNAs are dysregulated in many cancers, including PCa [5,6,28,29,30,31,32,33,34,35]. MiRNAs can play either the role of an oncogene when they target tumor suppressor genes and similarly as tumor suppressors when they target oncogenes [5,6,28,29,30,31,32,33,34,35]. Dysregulation of miRNAs signatures are not rare but rather the rule of human cancer, including PCa [5,6,28,29,30,31,32,33,34,35]. Thus, miRNA profiling has been a potent tool in identifying predictive miRNA signatures associated with the progression of various cancers [5,6,28,29,30,31,32,33,34,35].

Based on miRNA target prediction algorithm tools, TARGETSCAN (http://www.targetscan.org/vert_72/) and miRDB (http://mirdb.org/), microRNA-4719 and miRNA-6756-5p have been predicted to target the 3’ untranslated region (3’UTR) of IL-24 mRNA. The present study aims to examine the expression, function, and molecular mechanisms of action of miR-4719 and miR-6756-5p targeting of IL-24 in PCa progression *in vitro*. We discovered that miR-4719 and miR-6756-5p are significantly overexpressed in CRPC cell lines and particularly in African American men (AAM). Furthermore, IL-24 mRNA is decreased in all PCa cells compared to normal prostate epithelial cells and we demonstrate that inhibition of miR-4719 and miR-6756-5p increases IL-24 expression and significantly inhibits proliferation and migration of CRPC cell lines.

These discoveries of novel miRNA-based targeting of IL-24 may improve understanding of the molecular mechanisms in the development of CRPC and provide opportunities to explore clinical applications of miR-4719 and miR-6756-5p in PCa. In addition, understanding the mechanism in which IL-24 mRNA is stabilized could lead to the development of therapeutic strategies that prolong IL-24 mRNA half-life enabling enhanced killing in tumor cells. 

## 2. Results

### 2.1. MicroRNA-4719 and MicroRNA-6756-5p Are Significantly Overexpressed in Castration-Resistant Prostate Cancer (CRPC) Cells

**Figure 1:**
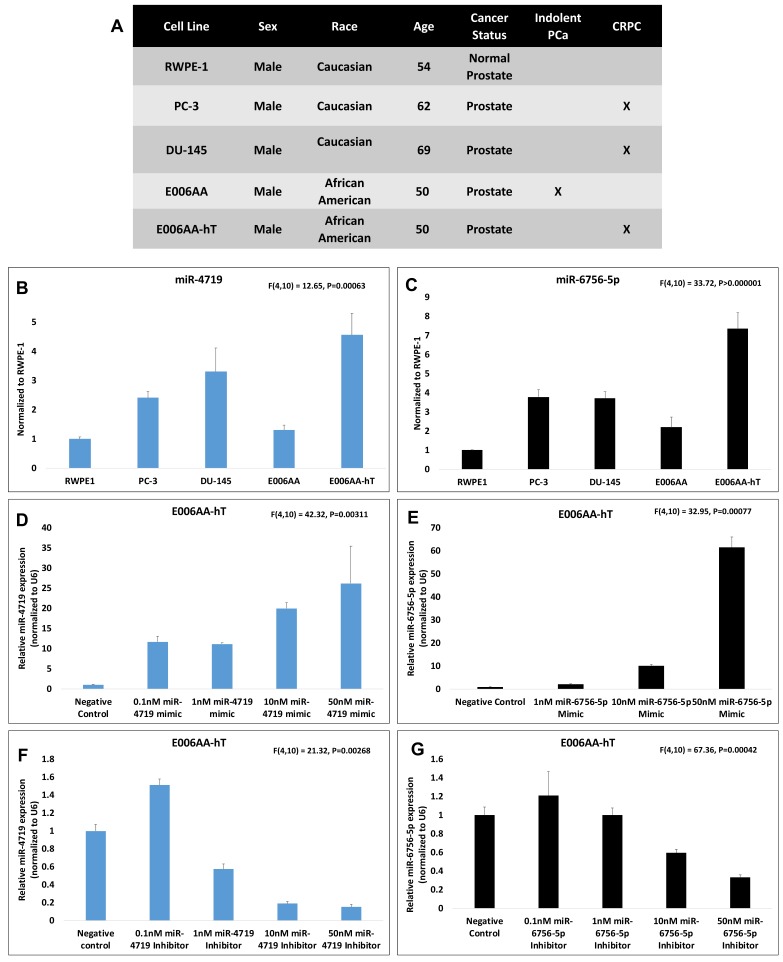
miR-4719 and miR-6756-5p are overexpressed in PCa cells and miR-4719 and miR-6756-5p inhibitors and mimics affect miR-4719 and miR-6756-5p expression. (**A**) Characterization of human cell lines used. qRT-PCR experiments were performed in quadruplicates. (**B**,**C**) MiR-4719 and miR-6756-5p levels were measured in DU-145, PC-3, E006AA, E006AA-hT and RWPE-1 cell lines. (**D**–**G**) A dose‒response test of the effect of a transiently transfected inhibitor and a mimic of miR-4719 and miR-6756-5p on miR-4719 and miR-6756-5p expression was performed on and measured against a 50 nM non-targeting negative control. Data are presented as mean ± standard error of the mean (SEM). Statistical differences were determined with one-way ANOVA; All the criterions for significance was set at *p* < 0.05.

Based on the predicted results from two miRNA molecular target prediction algorithms, TARGETSCAN and miRDB, we identified miR-4719 and miR-6756-5p as putative regulators of IL-24. These two microRNAs have never been studied in their regulation of IL-24 nor are there any known reports on the roles of miR-4719 and miR-6756-5p in PCa. To investigate the expression profiles, functional roles, and the molecular mechanisms of miR-4719 and miR-6756-5p as regulators of IL-24 in PCa biology, our work compares five different PCa cell lines, modeling different clinical characteristics of PCa, including E006AA (indolent PCa), E006AA-hT (CRPC), DU-145 (CRPC), and PC-3 (CRPC), in a non-tumorigenic prostate epithelial cell line, RWPE1 (Figure 1A).

qRT-PCR analysis shows that miR-4719 and miR-6756-5p are both significantly overexpressed in all PCa cell lines (by >2-fold) compared to the normal prostate epithelial cell line, RWPE-1 (Figure 1B,C). We observed that both miR-4719 (by at least 50%) and miR-6756-5p (>2-fold) are higher in CRPC cell lines compared to the indolent E006AA PCa cell line, indicating their gain may be an early event in PCa progression (Figure 1A,B). Additionally, both miR-4719 and miR-6756-5p expression were higher by (>3-fold) in the CRPC cell line E006AA-hT compared to indolent cell line-E006AA (Figure 1A,B). Interestingly, miR-4719 and miR-6756-5p is more significantly overexpressed in the CRPC of African-American men (AAM) (E006AA-hT) compared to aggressive PCa cell lines for Caucasian men (CM) (PC-3 and DU-145) (Figure 1A,B).

To elucidate the functional roles of miR-4719 and miR-6756-5p expression in PCa, commercially available synthetic oligonucleotide mimics of miR-4719 and miR-6756-5p (miR-4719 mimic and miR-6756-5p mimic), synthetic oligonucleotide inhibitors of miR-4719 and miR-6756-5p (miR-4719 inhibitor and miR-6756-5p inhibitor), or a synthetic non-targeting negative control oligonucleotide (negative control) were transfected into the cells using Lipofectamine^®^ RNAiMAX. A dose‒response experiment analyzed using qRT-PCR confirmed that the miR-4719 mimic and miR-6756-5p mimic increases endogenous expression of miR-4719 and miR-6756-5p, respectively, in a dose dependently fashion (Figure 1D,E). Similarly, the miR-4719 inhibitor and miR-6756-5p inhibitor decreases endogenous miR-4719 and miR-6756-5p expression, respectively, in a dose-dependent fashion (Figure 1F,G). A 50-nM concentration of both the mimics and inhibitors of miR-4719 and miR-6756-5p showed maximal specific effect on miR-4719 and miR-6756-5p expression. Consequently, the 50 nM dose was used to determine the roles of miR-4719 and miR-6756-5p in regulating proliferation and migration in CRPC cells.

### 2.2. IL-24 Is Downregulated in All Prostate Cancer Cells and microRNA-4719 and microRNA-6756-5p Targets IL-24

**Figure 2:**
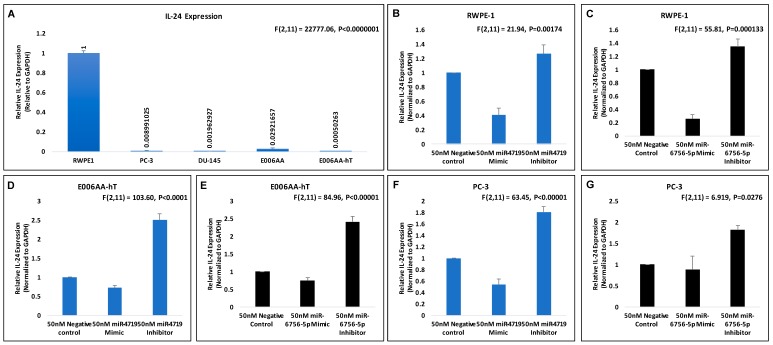
miR-4719 and miR-6756-5p targets IL-24. qRT-PCR experiments were performed in quadruplicates. (**A**–**G**) Effects of 50 nM concentration of microRNA mimics and inhibitors of miR-4719 and miR-6756-5p on IL-24 mRNA expression in the RWPE-1, PC-3 and the E006AA-hT PCa cell lines. Data are presented as mean ± standard error of the mean. Statistical differences were determined with one-way ANOVA. All the criterions for significance was set at *p* < 0.05.

Using our panel of PCa cell lines, we discovered that IL-24 expression is downregulated in aggressive CRPC cell lines compared to indolent PCa and the normal prostate epithelial cell line, RWPE-1 (Figure 2A). Our data reveals that IL-24 expression is lower in the CRPC cell lines PC-3, DU-145 and E006AA-hT cell lines when compared to its indolent counterpart, E006AA (Figure 2A). This suggests that loss of IL-24 is associated with the progression to CRPC.

We next investigated if IL-24 may possibly be a molecular target of miR-4719 and miR-6756-5p. Consequently, we assessed the effect of the microRNA mimics (overexpression) and inhibitors (loss) of miR-4719 and miR-6756-5p on IL-24 mRNA expression in the RWPE-1, E006AA-hT and PC-3 PCa cell lines (Figure 2B–G). qRT-PCR analysis show inhibition of miR-4719 and miR-6756-5p significantly increases the expression of IL-24 in RWPE-1 cells (~20%), E006AA-hT cells (~2.5 fold) and PC-3 cells (nearly 2-fold) compared to the negative control. In contrast, overexpression of miR-4719 and miR-6756-5p reveals significant decrease in IL-24 expression. Therefore, our findings indicate that miR-4719 and miR-6756-5p both regulate IL-24 expression in PCa cells. Strategies to inhibit miR-4719 and miR-6756-5p expression to increase IL-24 may have therapeutic efficacy in PCa.

### 2.3. Loss of miR-4719 and miR-6756-5p Significantly Inhibits Proliferation in CRPC Cells

**Figure 3:**
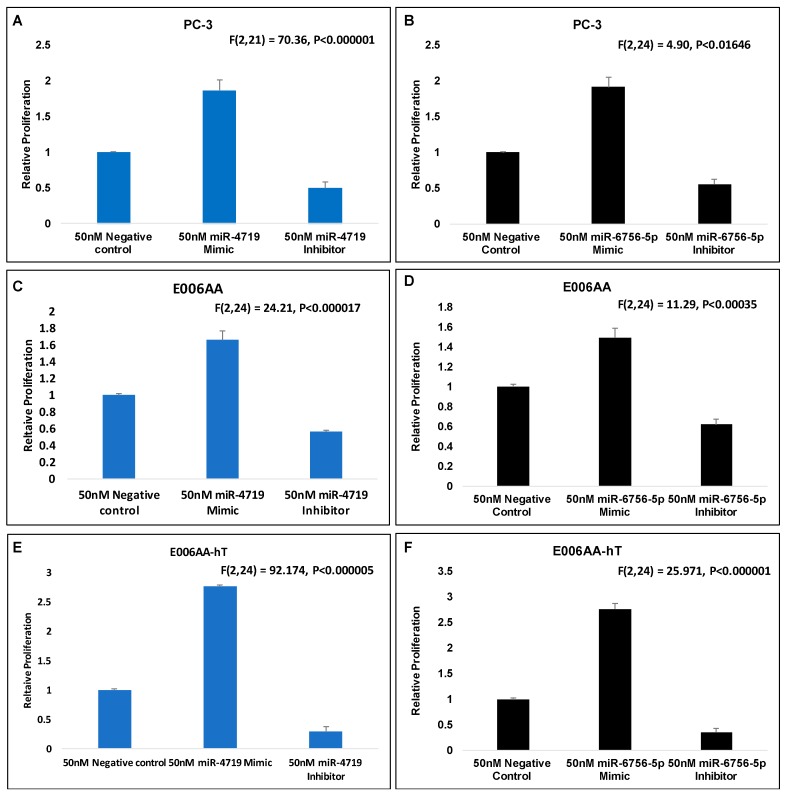
miR-4719 and miR-6756-5p loss significantly inhibits proliferation in CRPC. Transfection of a mimic or inhibitor of miR-4719 and miR-6756-5p was performed. (**A**–**F**) Effect of overexpression of miR-4719 and miR-6756-5p and inhibition of miR-4719 and miR-6756-5p expression on proliferation of PCa cells assessed using the MTT proliferation assay. Statistical differences were determined with one-way ANOVA. Data are presented as mean ± standard error of the mean. All the criterions for significance was set at *p* < 0.05.

We next compared the effects of inhibition and overexpression of miR-4719 and miR-6756-5p on PCa cell proliferation. MTT assays revealed that inhibition of miR-4719 and miR-6756-5p significantly decreases proliferation by >40%, while overexpression of miR-4719 and miR-6756-5p increases proliferation >50% in PC-3, E006AA and E006AA-hT PCa cell lines (Figure 3). Notably, we observed that overexpression of miR-4719 and miR-6756-5p increased proliferation most in CRPC cells (PC-3 and E006AA-hT, >2-fold) compared to benign PCa cells (E006AA, ~50%). Conversely, the inhibition of miR-4719 and miR-6756-5p decreased proliferation most in CRPC cells (PC-3 and E006AA-hT, >50%) compared to benign PCa cells (E006AA, ~35%). Inhibition of both miRNAs further decreased the proliferation of the CRPC E006AA-hT cell line by an extra 40%, compared to indolent E006AA cell line. In contrast, overexpression of miR-4719 and miR-6756-5p further increased the proliferation of the CRPC E006AA-hT by an at least 50%, compared to the indolent E006AA.

Interestingly, overexpression of miR-4719 and miR-6756-5p led to an even higher proliferative rate in the CRPC AAM PCa cell line E006AA-hT by at least 30% compared to the proliferative rate of the CRPC CM PCa cell line, PC-3. Similarly, loss of miR-4719 and miR-6756-5p led to a more reduced proliferative rate in the CRPC AAM PCa cell line E006AA-hT by at least 20% compared to the proliferative rate of the CRPC CM PCa cell line, PC-3. Overall, the data demonstrate that loss of miR-4719 and miR-6756-5p significantly inhibited cellular proliferation in CRPC.

### 2.4. Loss of miR-4719 and miR-6756-5p Significantly Inhibits Migration in CRPC Cells

**Figure 4:**
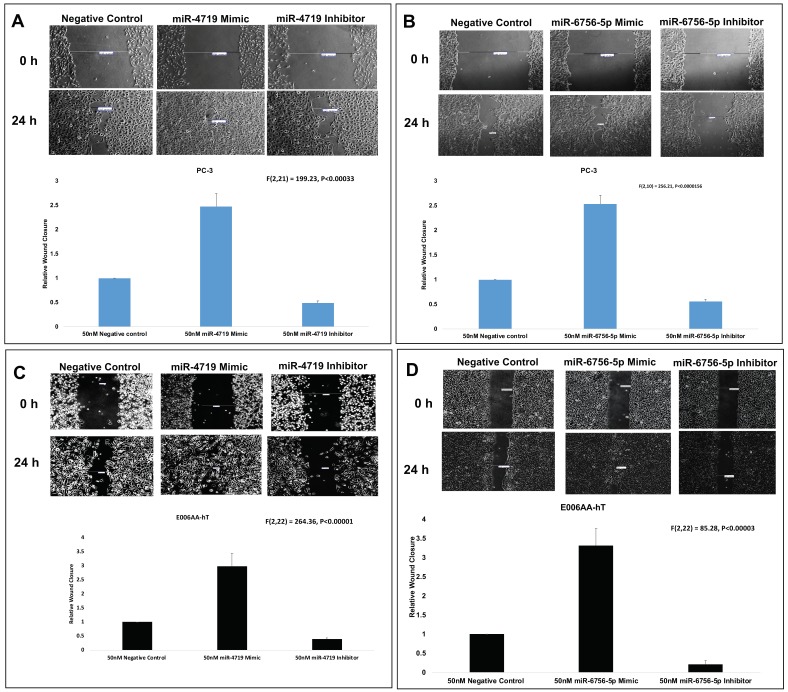
miR-4719 and miR-6756-5p loss significantly inhibits migration in CRPC. Transfection of a mimic or inhibitor of miR-4719 and miR-6756-5p was performed. (**A**–**D**) Effect of overexpression of miR-4719 & miR-6756-5p and inhibition of miR-4719 & miR-6756-5p expression on cell migration assessed using the wound healing assay. Statistical differences were determined with one-way ANOVA. Data are presented as mean ± standard error of the mean. All the criterions for significance was set at *p* < 0.05.

Lastly, wound healing assays were performed to assess effect of the mimics and inhibitors of miR-4719 and miR-6756-5p on migration of the CRPC PC-3 and E006AA-hT cell lines (Figure 4). Wound healing assays reveal that inhibition of miR-4719 and miR-6756-5p reduced migration by approximately 50% compared to the negative control of the CM cell line, PC-3 (Figure 4A,B). We observed that inhibition of miR-4719 and miR-6756-5p more effectively reduced the migration of CRPC E006AA-hT cells compared to the negative control, by ~60% and ~80%, respectively (Figure 4C,D). Strikingly, overexpression of miR-4719 and miR-6756-5p increased the migratory capacity of both CRPC cell lines, in PC-3 by greater than 2-fold and in E006AA-hT by at least 3-fold (Figure 4A–D). Taken together, loss of miR-4719 and miR-6756-5p inhibited proliferation and migration in CRPC cell lines.

## 3. Discussion

The chief obstacle in detecting and treating prostate cancer (PCa) is understanding the molecular mechanisms involved in the progression of indolent tumors to lethal castrate-resistant prostate cancer (CRPC) [1,2,7]. Understanding the molecular mechanisms is essential for the discovery of robust prognostic markers that can identify patients with the greatest risk of relapse and optimize management strategies to control PCa progression [1,2,7,9]. Thus, in cancer biology, microRNAs (miRNAs) studies have become important for the discovery of miRNA-based diagnostic, prognostic and theranostic biomarkers [5,6,28,29,30,31,32,33,34,35]. Changes in miRNA levels can provide critical information on a disease’s molecular signature status through the course of treatment and recurrence without the need for a biopsy [5,6,28,29,30,31,32,33,34,35].

Here, we have presented the rationale for investigating the novel roles of microRNA-4719 and microRNA-6756-5p in respect to Interleukin-24 (IL-24) in PCa. The miRNA mechanisms regulating IL-24 mRNA or protein levels have not been fully delineated in the progression of PCa. When overexpressed, IL-24 kills PCa cells through several pathways. IL-24 can cause mitochondrial dysfunction, reactive oxygen species production, and calcium mobilization leading to apoptosis in PCa cell lines [12]. IL-24 can also downregulate anti-apoptotic proteins such as Bcl-2 and Bcl-xL and produce of tumor-suppressing ceramides leading to endoplasmic reticulum stress, autophagy, and apoptosis in PCa [11,12,13,14,15,16,36]. In mouse PCa xenografts, IL-24 decreases the levels of secreted soluble clusterin (sCLU) protein, which is linked to resistance to chemotherapy and radiation and hormone therapies, leading to reduced tumor growth and angiogenesis [37]. Supporting these studies, IL-24 has been found to reduce factors of stemness such as migration, invasion, colony formation and inhibit the expression of SOX2 transcription factor, CD44 cell-surface glycoprotein and ATP Binding Cassette Subfamily G Member 2 (ABCG2) compared to other cytokines, IL-3, IL-6 and IL-11 in PCa cells [26]. In combination with ionizing radiation, IL-24 is also able to induce apoptosis in PCa cells overexpressing Bcl-2 and Bcl-xL proteins that would normally exhibit resistance to IL-24 treatment alone [17]. Combination treatment of Sabutoclax, an inhibitor of prosurvival myeloid cell leukemia (Mcl-1) protein, with IL-24 also sensitizes prostate cancer tumors to IL-24-mediated apoptosis [18]. 

In terms of IL-24 expression regulation in cancer cells, two studies have demonstrated that IL-24 mRNA regulation occurs at the 3’ untranslated region (3’UTR) end via activation of p38MAPK [13,38]. In addition, miRNA-205, which is silenced in human prostate cell lines, has been shown to directly target the IL-24 promoter to induce gene expression in human PCa and oral cancer cells [25,38]. Furthermore, IL-24 mRNA has been reported to have a relatively short half-life (~20 min) due to the presence of three AU-rich elements (ARE-sequences), which are targets for RNA-binding proteins leading to mRNA destabilization and degradation [38].

**Figure 5:**
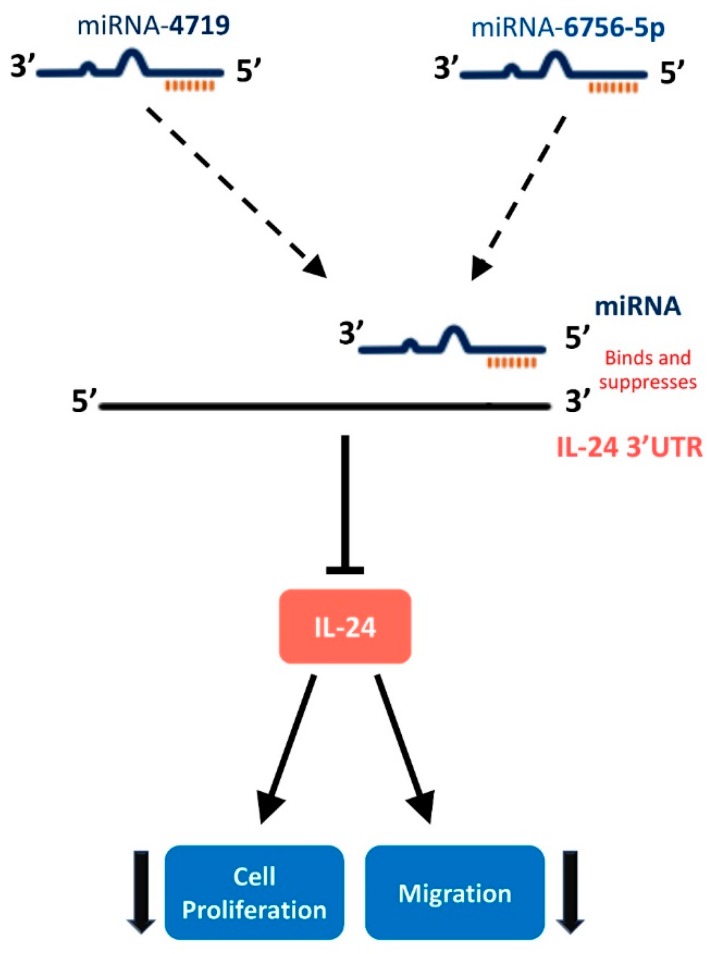
Hypothesized pathway of microRNA-4719 and microRNA-6756-5p targeting IL-24 to regulate cellular proliferation and migration in CRPC cell lines. miR-4719 and miR-6756-5p bind to IL-24 3′UTR leading to destabilization of IL-2 mRNA, a decrease in IL-24 protein production, and an increase in CRPC cell proliferation and migration.

In this study, we describe for the first time an IL-24/miR-4719 and IL-24/miR-6756-5p regulatory pathway in PCa which has important clinical implications as miRNAs possess several key features that make them attractive PCa biomarkers (Figure 5). We discovered that the expression of miR-4719 and miR-6756-5p is significantly overexpressed in PCa cells (Figure 1). Furthermore, both microRNAs demonstrate differential expression between indolent and CRPC. We also demonstrate expression of miR-4719 and miR-6756-5p in PCa cells significantly increases cancer cell proliferation and migration (Figure 3 and Figure 4). As predicted, we show that miRNA-4719 and miRNA-6756-5p inhibitors also increase the expression of IL-24 mRNA and also significantly inhibit PCa and CRPC proliferation and migration (Figure 2, Figure 3 and Figure 4). As miRNAs are known to be involved in the destabilization of mRNA thus, reducing expression of proteins such as tumor suppressors, and we show that miRNA-4719 and miRNA-6756-5p decreases IL-24 expression, it is possible that miRNA-4719 and miRNA-6756-5p are involved in post-transcriptional modification of IL-24 by destabilizing IL-24 3’UTR, thus, shortening IL-24’s half-life. 

To increase IL-24’s specific killing effect, miRNA-4719 and miRNA-6756-5p oligonucleotide inhibitors could be used in conjunction with IL-24 treatment to increase endogenous production of IL-24 and thus, increase apoptosis in cancer cells. It has been shown that miRNAs can simultaneously modulate several cancer-relevant gene pathways and can be exploited to increase the sensitivity of tumor cells to conventional anticancer agents [29,31,33,39,40,41]. Due to the potential to be one-hit multi-target therapeutic agents against PCa, miRNAs are interesting drug candidates [28,31,39,42]. Furthermore, our functional experiments clearly demonstrate a mechanism by which miR-4719 and miR-6756-5p regulate key cellular processes that are dysregulated in the development and progression of PCa thus, strategies to reduce miR-4719 and miR-6756-5p in PCa may have therapeutic value in CRPC. In terms of diagnostic significance, the increased levels of miR-4719 and miR-6756-5p expression in CRPC cells compared to normal prostate epithelial cells indicates that both miRNAs may potentially serve as biomarkers to risk stratify PCa. Further studies using clinical samples are needed to test the potential of miR-4719 and miR-6756-5p as biomarkers, however. Interestingly, a recent study showed that circulating miRNA-6756-5p along with miR-1246, and miR-8073 were successfully used as biomarkers to detect breast cancer with a 97.1% accuracy [43]. Along with our work, this suggests that miRNA-6756-5p could be a useful biomarker for detecting various cancer cell types. 

Along with studying how miRNA-4719 and miRNA-6756-5p affects IL-24 expression, we also investigated the impact of racial disparity in PCa since African American men (AAM) have a 2-fold higher chance of getting aggressive PCa and disparities in tumor aggressiveness remain after controlling for social determinants [32,44,45,46,47,48]. To address this high mortality, effective early detection and therapeutic strategies are needed [32,44,45,46,47,48]. In this regard, we discovered that miR-4719 and miR-6756-5p seems to directly correlate with aggressive PCa cell lines and is differentially expressed between Caucasian men (CM) and AAM cell lines tested in our study (Figure 1B,C). Moreover, miR-4719 and miR-6756-5p significantly inhibited IL-24 expression more potently in the AAM cell line compared to the CM cell lines (Figure 2D–G). The data suggests that miR-4719 and miR-6756-5p may play a role in explaining the disproportionately increased aggressiveness of PCA in AAM. A limitation of our study is the small sample size. The problem lies in the fact that the majority of established PCa cell lines are derived from Caucasian men (CM) patients. To date, there are only four authentic and spontaneously transformed African American men (AAM) PCa cell lines [47,48]. These include E006AA (primary PCa cell line), E006AA-ht (a subline of E006AA), MDA-PCa2a and MDA-PCa2b [47,48]. In our study, we were able to compare these two proven cell lines, (E006AA and E006AA-ht) derived from AAM with different clinical characteristics which are clinically relevant and compared them against three cell lines from CM with different clinical characteristics (RWPE-1, PC-3, and DU-145). In future studies, we intend to investigate miR-4719 and miR-6756-5p in prostate cancer tissue samples derived from both AAM and CM. The discovery of a miRNA biomarker that can display differential expression between races is of great significance for the development and optimization of miR-4719 and miR-6756-5p-based therapeutic strategies for personalized treatment. 

In conclusion, this is the first report to show miR-4719 and miR-6756-5p as potential regulators of IL-24 mRNA expression and as candidate biomarkers for risk stratification of PCa, as well as help explain the racial disparity of PCa. Loss of miR-4719 and miR-6756-5p significantly inhibited proliferation and migration in CRPC cell lines via targeting of IL-24. Consequently, these *in vitro* studies also demonstrate the potential of miR-4719 and miR-6756-5p serving as both diagnostic biomarkers and as therapeutics for CRPC.

## 4. Materials and Methods

### 4.1. Cell Culture

Androgen-dependent RWPE-1 cells were cultured in keratinocyte serum-free medium (SFM) supplemented with 0.05 mg/mL bovine pituitary extract (BPE), 5 ng/mL epidermal growth factor (EGF) and 1% penicillin-streptomycin. Androgen-independent PC-3 cells were cultured in F-12K medium supplemented with 10% fetal bovine serum and 1% penicillin/streptomycin. Androgen-independent DU-145 cells were cultured in MEM supplemented with 10% fetal bovine serum and 1% penicillin/streptomycin. Androgen-independent E006AA and E006AA-hT were cultured in DMEM supplemented with 1% fetal bovine serum and 1% penicillin/streptomycin. RWPE-1, PC-3, DU-145, Eoo6AA and E006AA-hT cell lines were purchased from the American Type Culture Collection (Manassas, VA, USA). These cells were cultured at 37 °C in 5% CO_2_, under a humidified atmosphere.

### 4.2. Transfections of Oligonucleotides

Cells were seeded in six-well plates. After reaching 60–70% confluence, the medium is replaced with Opti-MEM (Thermo Fisher Scientific Inc.; Wilmington, DE, USA) and cells are transfected with either a 50 nM non-targeting negative control oligonucleotide (MISSION^®^ Synthetic microRNA Negative Control, human, product# NCSTUD001), 50 nM miR-4719 (MISSION^®^ microRNA Mimic, human, product# HMI1756), miR-6756-5p oligonucleotide mimic (MISSION^®^ microRNA Mimic, human, product# HMI2362), 50 nM miR-4719 (MISSION^®^ microRNA inhibitor, human, product# HSTUD1756) or miR-6756-5p oligonucleotide inhibitor (MISSION^®^ Synthetic microRNA Inhibitor, human, product# HSTUD00363) (Sigma-Aldrich, St. Louis, MO, USA), using Lipofectamine RNAiMAX (Thermo Fisher Scientific Inc.; Wilmington, DE, USA) according to the manufacturer’s instructions. Transfected cells are then incubated at 37 °C for a total duration of 24 h before cells are lysated.

### 4.3. RNA Isolation and Quantitative Real-Time Polymerase Chain Reaction (qRT-PCR) Analysis

MicroRNA isolation was performed using Invitrogen™ *mir*Vana™ miRNA Isolation Kit, with phenol, (Catalog Number: AM1560) according to manufacturer instructions. cDNA and qRT-PCR were performed using Qiagen miScript II RT Kit (50) (Catalog Number: 218161) and Qiagen miScript SYBR^®^ Green PCR Kits (Catalog Number: 218073) (Qiagen, Germantown, MD, USA), respectively. The following primers were used: RNU6_B12 snRNA primers were purchased from Qiagen (Hs_RNU6-2_11 miScript Primer Assay RNU6-6P RNA, U6 small nuclear 6, pseudogene: Product #218300, Catalog Number: MS00033740). Hs_miR-4719_1 miScript Primer Assay and miR-6756-5p miScript Primer Assay were purchased from Qiagen (hsa-miR-4719: Product # 218300, Catalog Number: MS00033740) and (has-miR-6756-5p: Product # 218300, Catalog number: MS00046606). Total RNA was isolated by using Qiagen RNeasy Mini Kit (50) (Catalog Number 74104). Reverse transcription was performed on 1 µg of total RNA with an oligo(dT) primer using Qiagen QuantiTect Rev. Transcription Kit (50) (Catalog Number: 205311). cDNA corresponding to 20 ng of total RNA was amplified for 35 cycles by PCR with specific primers using Thermo Fisher Applied Biosystems™SYBR™ Green PCR Master Mix (Catalog Number: 4309155). The following primers were used: IL-24, FORWARD 5′-TTCTCTGGAGCCAGGTATC-3′, REVERSE 5-’TAGAATTTCTGCATCCAGGT-3, GAPDH, FORWARD 5′-AGCTTGTCATCAATGGAAAT-3, REVERSE 5′-CTTCACCACCTTCTTGATGT-3′.

### 4.4. MTT Assay

Briefly, cells were plated in 96-well dishes ((1 × 10^3^)/well), with three replicates for each group and allowed to attach for 24 h prior to treatment(s). Cell growth and viable cell numbers were monitored by 3-(4,5 dimethylthiazol-2-yl)-2,5-diphenyltetrazolium bromide (MTT) staining. The optical density (OD) value was measured at 595 nm with a microplate reader (Bio-Rad, Hercules, CA, USA).

### 4.5. Wound Healing Assay

First, 1 × 10^4^ cells were seeded into six-well plates. At 90% confluency, the cell monolayer was wounded with a 200-μL pipette, washed with PBS, and the medium replaced with OPTI-MEM with treatments of a 50 nM non-targeting negative control oligonucleotide, 50 nM miR-4719 oligonucleotide mimic, miR-6756-5p oligonucleotide mimic, 50 nM miR-4719 oligonucleotide inhibitor, or miR-6756-5p oligonucleotide inhibitor using Lipofectamine RNAiMAX according to the manufacturer. Images were taken 24 h later. Images were taken using Motic Images Plus 2.0 Software (Motic; Richmond, BC, Canada) from three experiments and were analyzed for percentage cell-covered area.

### 4.6. Statistical Analysis

Data were collected from at least three independent experiments. All results are presented as mean ± standard error of the mean (SEM). Unless otherwise indicated, analysis of statistical significance of differences between groups was performed using two-tailed Student’s *t*-test, and only values with *p* < 0.05 were deemed significant. For comparison of variables, a Student’s *t*-test or analysis of variance (ANOVA) test were used for analysis of each set of continuous and categorical data. Statistical differences in the relative miRNA expression profiles were determined with one-way analysis of variance (ANOVA) using the SPSS Statistics software (http://www-01.ibm.com/software/analytics/spss/) on normalized data. *p* < 0.05 was considered significant.

## References

[1] Katsogiannou M., Ziouziou H., Karaki S., Andrieu C., Henry de Villeneuve M., Rocchi P. (2015). The hallmarks of castration-resistant prostate cancers. Cancer Treat. Rev..

[2] Parnes H.L., House M.G., Tangrea J.A. (2013). Prostate cancer prevention: Strategies for agent development. Curr. Opin. Oncol..

[3] Siegel R.L., Miller K.D., Jemal A. (2017). Cancer statistics, 2017. CA Cancer J. Clin..

[4] DeSantis C.E., Siegel R.L., Sauer A.G., Miller K.D., Fedewa S.A., Alcaraz K.I., Jemal A. (2016). Cancer statistics for African Americans, 2016: Progress and opportunities in reducing racial disparities. CA Cancer J. Clin..

[5] Luu H.N., Lin H.Y., Sørensen K.D., Ogunwobi O.O., Kumar N., Chornokur G., Phelan C., Jones D., Kidd L.C., Batra J. (2017). miRNAs associated with prostate cancer risk and progression. BMC Urol..

[6] Wadhwa B., Dumbre R. (2016). Achieving resistance specificity in prostate cancer. Chem. Biol. Interact..

[7] Wiklund F. (2010). Prostate cancer genomics: Can we distinguish between indolent and fatal disease using genetic markers?. Genome Med..

[8] Moyer V.A. (2012). Screening for prostate cancer: U.S. preventive services task force recommendation statement. Ann. Intern. Med..

[9] Obinata D., Takayama K., Takahashi S., Inoue S. (2017). Crosstalk of the androgen receptor with transcriptional collaborators: Potential therapeutic targets for castration-resistant prostate cancer. Cancers.

[10] Wei J.T., Feng Z., Partin A.W., Brown E., Thompson I., Sokoll L., Chan D.W., Lotan Y., Kibel A.S., Busby J.E. (2014). Can urinary PCA3 Supplement PSA in the early detection of prostate cancer?. J. Clin. Oncol..

[11] Bhutia S.K., Dash R., Das S.K., Azab B., Su Z.Z., Lee S.G., Grant S., Yacoub A., Dent P., Curiel D.T. (2010). Mechanism of autophagy to apoptosis switch triggered in prostate cancer cells by antitumor cytokine melanoma differentiation-associated gene 7/interleukin-24. Cancer Res..

[12] Do W., Herrera C., Mighty J., Shumskaya M., Redenti S.M., Sauane M. (2013). Sigma 1 receptor plays a prominent role in IL-24-induced cancer-specific apoptosis. Biochem. Biophys. Res. Commun..

[13] Sauane M., Su Z.-Z., Gupta P., Lebedeva I.V., Dent P., Sarkar D., Fisher P.B. (2008). Autocrine regulation of mda-7/IL-24 mediates cancer-specific apoptosis. Proc. Natl. Acad. Sci. USA.

[14] Lebedeva I.V., Sarkar D., Su Z.Z., Kitada S., Dent P., Stein C.A., Reed J.C., Fisher P.B. (2003). Bcl-2 and Bcl-x(L) differentially protect human prostate cancer cells from induction of apoptosis by melanoma differentiation associated gene-7, mda-7/IL-24. Oncogene.

[15] Sauane M., Su Z.Z., Dash R., Liu X., Norris J.S., Sarkar D., Lee S.G., Allegood J.C., Dent P., Spiegel S. (2010). Ceramide plays a prominent role in MDA-7/IL-24-induced cancer-specific apoptosis. J. Cell. Physiol..

[16] Tian H., Wang J., Zhang B.F., Di J.H., Chen F.F., Li H.Z., Li L.T., Pei D.S., Zheng J.N. (2012). MDA-7/IL-24 induces Bcl-2 denitrosylation and ubiquitin-degradation involved in cancer cell apoptosis. PLoS ONE.

[17] Su Z.Z., Lebedeva I.V., Sarkar D., Emdad L., Gupta P., Kitada S., Dent P., Reed J.C., Fisher P.B. (2006). Ionizing radiation enhances therapeutic activity of mda-7/IL-24: Overcoming radiation- and mda-7/IL-24-resistance in prostate cancer cells overexpressing the antiapoptotic proteins bcl-xLor bcl-2. Oncogene.

[18] Dash R., Azab B., Quinn B.A., Shen X., Wang X.-Y., Das S.K., Rahmani M., Wei J., Hedvat M., Dent P. (2011). Apogossypol derivative BI-97C1 (Sabutoclax) targeting Mcl-1 sensitizes prostate cancer cells to mda-7/IL-24-mediated toxicity. Proc. Natl. Acad. Sci. USA.

[19] Lebedeva I.V., Su Z.Z., Sarkar D., Kitada S., Dent P., Waxman S., Reed J.C., Fisher P.B. (2003). Melanoma differentiation associated Gene-7, mda-7/Interleukin-24, induces apoptosis in prostate cancer cells by promoting mitochondrial dysfunction and inducing reactive oxygen species. Cancer Res..

[20] Yang J., Yin H., Wei Y., Fang L., Chai D., Zhang Q., Zheng J., Yin H., Yang J., Zheng J. (2019). Tumor-penetrating peptide enhances antitumor effects of IL-24 against prostate cancer. Transl. Oncol..

[21] Majid S., Dar A.A., Saini S., Yamamura S., Hirata H., Tanaka Y., Deng G., Dahiya R. (2010). MicroRNA-205-directed transcriptional activation of tumor suppressor genes in prostate cancer. Cancer.

[22] Yu D.-D., Zhong Y.-L., Li X.-R., Li Y.-Q., Li X.-L., Cao J., Fan H.-J., Yuan Y., Ji Z.-Y., Qiao B.-P. (2015). ILs-3, 6 and 11 increase, but ILs-10 and 24 decrease stemness of human prostate cancer cells in vitro. Oncotarget.

[23] Dash R., Richards J.E., Su Z.Z., Bhutia S.K., Azab B., Rahmani M., Dasmahapatra G., Yacoub A., Dent P., Dmitriev I.P. (2010). Mechanism by which Mcl-1 regulates cancer-specific apoptosis triggered by mda-7/IL-24, an IL-10-related cytokine. Cancer Res..

[24] Mao L., Ding M., Xu K., Pan J., Yu H., Yang C. (2018). Oncolytic adenovirus harboring Interleukin-24 improves chemotherapy for advanced prostate cancer. J. Cancer.

[25] Sauane M., Lebedeva I.V., Su Z.Z., Choo H.T., Randolph A., Valerie K., Dent P., Gopalkrishnan R.V., Fisher P.B. (2004). Melanoma differentiation associated Gene-7/Interleukin-24 promotes tumor cell-specific apoptosis through both secretory and nonsecretory pathways. Cancer Res..

[26] Bhutia S.K., Das S.K., Azab B., Dash R., Su Z.Z., Lee S.G., Dent P., Curiel D.T., Sarkar D., Fisher P.B. (2011). Autophagy switches to apoptosis in prostate cancer cells infected with melanoma differentiation associated gene-7/interleukin-24 (mda-7/IL-24). Autophagy.

[27] Iorio M.V., Croce C.M. (2012). MicroRNA dysregulation in cancer: Diagnostics, monitoring and therapeutics. A comprehensive review. EMBO Mol. Med..

[28] Lu J., Getz G., Miska E.A., Alvarez-Saavedra E., Lamb J., Peck D., Sweet-Cordero A., Ebert B.L., Mak R.H., Ferrando A.A. (2005). MicroRNA expression profiles classify human cancers. Nature.

[29] MacFarlane L.A., Murphy P.R. (2010). MicroRNA: Biogenesis, function and role in cancer. Curr. Genom..

[30] Cannistraci A., Di Pace A.L., De Maria R., Bonci D. (2014). MicroRNA as new tools for prostate cancer risk assessment and therapeutic intervention: Results from clinical data set and patients’ samples. BioMed Res. Int..

[31] Jones J., Grizzle W., Wang H., Yates C. (2013). MicroRNAs that affect prostate cancer: Emphasis on prostate cancer in African Americans. Biotech Histochem..

[32] Iorio M.V., Croce C.M. (2009). MicroRNAs in cancer: Small molecules with a huge impact. J. Clin. Oncol..

[33] Volinia S., Calin G.A., Liu C.-G., Ambs S., Cimmino A., Petrocca F., Visone R., Iorio M., Roldo C., Ferracin M. (2006). A microRNA expression signature of human solid tumors defines cancer gene targets. Proc. Natl. Acad. Sci. USA.

[34] Ayub S.G., Kaul D., Ayub T. (2015). Microdissecting the role of microRNAs in the pathogenesis of prostate cancer. Cancer Genet..

[35] Bhutia S.K., Das S.K., Kegelman T.P., Azab B., Dash R., Su Z.Z., Wang X.Y., Rizzi F., Bettuzzi S., Lee S.G. (2012). MDA-7/IL-24 differentially regulates soluble and nuclear clusterin in prostate cancer. J. Cell. Physiol..

[36] Otkjaer K., Holtmann H., Kragstrup T.W., Paludan S.R., Johansen C., Gaestel M., Kragballe K., Iversen L. (2010). The p38 MAPK regulates IL-24 expression by stabilization of the 3′ UTR of IL-24 mRNA. PLoS ONE.

[37] Rupaimoole R., Slack F.J. (2017). MicroRNA therapeutics: Towards a new era for the management of cancer and other diseases. Nat. Rev. Drug Discov..

[38] Calin G.A., Sevignani C., Dumitru C.D., Hyslop T., Noch E., Yendamuri S., Shimizu M., Rattan S., Bullrich F., Negrini M. (2004). Human microRNA genes are frequently located at fragile sites and genomic regions involved in cancers. Proc. Natl. Acad. Sci. USA.

[39] Watahiki A., Wang Y., Morris J., Dennis K., O’Dwyer H.M., Gleave M., Gout P.W., Wang Y. (2011). MicroRNAs associated with metastatic prostate cancer. PLoS ONE.

[40] Karlou M., Tzelepi V., Efstathiou E. (2010). Therapeutic targeting of the prostate cancer microenvironment. Nat. Rev. Urol..

[41] Cui X., Li Z., Zhao Y., Song A., Shi Y., Hai X., Zhu W. (2018). Breast cancer identification via modeling of peripherally circulating miRNAs. PeerJ.

[42] Chung C.C., Hsing A.W., Yeboah E., Biritwum R., Tettey Y., Adjei A., Cook M.B., De Marzo A., Netto G., Tay E. (2014). A comprehensive resequence-analysis of 250 kb region of 8q24.21 in men of African ancestry. Prostate.

[43] Evans S., Metcalfe C., Ibrahim F., Persad R., Ben-Shlomo Y. (2008). Investigating Black-White differences in prostate cancer prognosis: A systematic review and meta-analysis. Int. J. Cancer.

[44] Hoffman R.M., Gilliland F.D., Eley J.W., Harlan L.C., Stephenson R.A., Stanford J.L., Albertson P.C., Hamilton A.S., Hunt W.C., Potosky A.L. (2001). Racial and ethnic differences in advanced-stage prostate cancer: The prostate cancer outcomes study. J. Natl. Cancer Inst..

[45] Koochekpour S., Willard S.S., Shourideh M., Ali S., Liu C., Azabdaftari G., Saleem M., Attwood K. (2014). Establishment and characterization of a highly tumorigenic African American prostate cancer cell line, E006AA-hT. Int. J. Biol. Sci..

[46] Koochekpour S., Maresh G.A., Katner A., Parker-Johnson K., Lee T.-J., Hebert F.E., Kao Y.S., Skinner J., Rayford W. (2004). Establishment and characterization of a primary androgen-responsive African-American prostate cancer cell line, E006AA. Prostate.

[47] Presley C.J., Raldow A.C., Cramer L.D., Soulos P.R., Long J.B., Yu J.B., Makarov D.V., Gross C.P. (2013). A new approach to understanding racial disparities in prostate cancer treatment. J. Geriatr. Oncol..

[48] Powell I.J., Bock C.H., Ruterbusch J.J., Sakr W. (2010). Evidence Supports a Faster Growth Rate and/or Earlier Transformation to Clinically Significant Prostate Cancer in Black Than in White American Men, and Influences Racial Progression and Mortality Disparity. J. Urol..

